# Integrated multi-omics and phenotypic validations reveal biocontrol mechanisms of *Bacillus velezensis* XM18-5 against potato common scab

**DOI:** 10.3389/fmicb.2026.1748645

**Published:** 2026-02-11

**Authors:** Xigang Wang, Jingjing An, Chengjin Guo, Jing Tian, Ruiqing Shen, Pei Zhao

**Affiliations:** Ningxia Key Laboratory of Plant Disease and Pest Control/Institute of Plant Protection, Ningxia Academy of Agriculture and Forestry Sciences, Yinchuan, China

**Keywords:** *Bacillus velezensis*, biocontrol, genomics, metabolomics, potato common scab

## Abstract

Potato common scab, caused by pathogenic Streptomyces species, is a devastating soil-borne disease that severely compromises potato yield and marketability. To develop effective biological control strategies and elucidate their molecular mechanisms, this study isolated a potent antagonistic strain, XM18-5, from disease-suppressive soil. Through a systematic approach integrating phenotypic assays, whole-genome sequencing, and non-targeted metabolomics, the strain was identified as *Bacillus velezensis*. *In vitro* assays demonstrated that XM18-5 exhibits significant antagonistic activity against *Streptomyces scabies* X-1 (60.72% inhibition rate), causing severe hyphal deformation and lysis as revealed by scanning electron microscopy. In pot experiments, XM18-5 treatment achieved a biocontrol efficacy of 70.90% against potato common scab and displayed broad-spectrum antifungal activity against ten other plant pathogens. Genome mining uncovered 12 biosynthetic gene clusters (BGCs) encoding diverse antimicrobial compounds, including surfactin, fengycin, and difficidin. Crucially, metabolomic analysis of the fermentation broth at the stationary phase provided direct material evidence for this genetic potential, identifying a specific chemical cocktail containing the lipopeptide Surfactin, the dipeptide antibiotic Bacilysin, and broad-spectrum antibiotics such as Erythromycin. Furthermore, metabolic pathway analysis revealed a significant upregulation of amino acid biosynthesis (e.g., valine, leucine, and phenylalanine), ensuring a robust precursor supply for these antimicrobial secondary metabolites. In conclusion, *B. velezensis* XM18-5 suppresses potato common scab through a synergistic mechanism driven by a genome-encoded, metabolically supported arsenal of antimicrobial compounds. This study provides a comprehensive understanding of the biocontrol mechanisms of XM18-5, establishing it as a promising candidate for bio-fertilizer development.

## 1 Introduction

Potato (*Solanum tuberosum* L.), the world’s fourth-largest food crop, is vital for global food security (Ahmadu et al., 2021). However, potato production is persistently challenged by various diseases, among which potato common scab, caused by several pathogenic Streptomyces species, is one of the most destructive soil-borne diseases worldwide (Sharma et al., 2024). The pathogen infects young tubers and secretes phytotoxins, such as thaxtomins, which interfere with host cell wall synthesis, leading to the formation of suberized scab lesions on the tuber surface. This severely reduces the commercial value and marketability of potatoes (Wanner and Kirk, 2015; Braun et al., 2017). Although traditional agronomic practices like crop rotation, use of resistant cultivars, and soil pH adjustment offer partial control, their effects are often limited and slow (Ghorbani et al., 2008). In terms of chemical control, specific and highly effective agents are still lacking, and long-term application of chemical pesticides can exacerbate pathogen resistance, disrupt soil micro-ecology, and pose food safety risks (Meena et al., 2020; Singh et al., 2020). Therefore, developing eco-friendly and efficient disease control strategies is an urgent need for the sustainable development of the potato industry.

Biological control, particularly the use of beneficial microorganisms to suppress pathogen growth, has emerged as a core strategy to replace chemical pesticides and achieve green agricultural production (Elnahal et al., 2022). Compared to traditional methods relying on phenotypic screening, modern biocontrol research increasingly focuses on discovering microbial resources with superior biocontrol potential from the source. In this context, the application of genomics has led to revolutionary breakthroughs. By performing whole-genome sequencing of candidate strains, researchers can accurately assess their genetic potential at an early screening stage. For example, bioinformatic tools like antiSMASH can rapidly predict the presence of biosynthesis gene clusters (BGCs) encoding antimicrobial substances (e.g., antibiotics, lipopeptides), while annotation against the CAZy database can identify genes for enzymes that degrade pathogen cell walls (Martinez-Vidales et al., 2024; Yang et al., 2024; Blin et al., 2025). This “inside-out” screening strategy significantly enhances the targeted discovery and success rate of identifying potent biocontrol strains, providing powerful technical support for the exploitation of microbial resources.

Among the vast array of biocontrol microorganisms, *Bacillus velezensis* is recognized as a “star” strain due to its ability to produce a rich diversity of secondary metabolites and exhibit broad-spectrum antimicrobial activity (Wang et al., 2021; Wang et al., 2024). Numerous studies have confirmed that the biocontrol capacity of *B. velezensis* stems from its diverse “chemical arsenal,” including lipopeptide families like surfactin, fengycin, and iturin, as well as polyketides (e.g., difficidin, macrolactin) and bacilysin (Wu et al., 2015; Fazle Rabbee and Baek, 2020). Although *B. velezensis* has been successfully used against various plant diseases, there are a limited number of reports on its application to control potato scab (Jiang et al., 2025). More importantly, most existing studies focus primarily on validating phenotypic effects, lacking in-depth investigations that systematically unravel the specific molecular mechanisms of antagonism against *Streptomyces scabies* at both the genomic and metabolomic levels.

Given the above, this study was initiated to screen and identify a potent antagonistic strain from a potato common scab-infested soil. We integrated phenotypic validation, whole-genome sequencing, and non-targeted metabolomics with the objectives to: (1) evaluate the practical biocontrol efficacy of strain XM18-5 against potato common scab; (2) reveal its antagonistic genetic potential through genome mining; and (3) identify the key active metabolites responsible for its function via metabolomic analysis. This work aims to provide new insights into the interaction mechanisms between *B. velezensis* and *S. scabies* and to offer a well-characterized candidate strain for the development of a novel microbial agent against potato common scab.

## 2 Materials and methods

### 2.1 Microorganisms, culture media, and reagents

The antagonistic strain *Bacillus velezensis* XM18-5 (accession number: CGMCC 25698) was isolated from the rhizosphere soil of a potato field affected by common scab in Malian Township, Xiji County, Ningxia, China. The strain was stored in LB broth containing 25% (v/v) glycerol at −80°C. The pathogen, *Streptomyces scabies* X-1, was isolated and preserved by our laboratory.

Luria-Bertani (LB) broth, LB agar, Oatmeal Agar (OMA), and Gause’s No. 1 synthetic agar medium were prepared according to the Manual of Plant Pathology Research Methods. The bacterial genomic DNA extraction kit was purchased from Solarbio Science & Technology Co., Ltd. (Cat#D1600). The physiological and biochemical identification kit (HBIG14) was obtained from Qingdao Haibo Biotechnology Co., Ltd. All other chemical reagents used were of analytical grade.

### 2.2 Isolation and screening of antagonistic strain

The antagonistic bacterium was isolated using the soil dilution plate method. One gram of air-dried soil was suspended in 9 mL of sterile water and serially diluted to 10^−4^, 10^−5^, 10^−6^, and 10^−7^. Aliquots of 100 μL from each dilution were spread onto OMA plates in triplicate. The plates were incubated at 30°C for 18–24 h. Colonies with distinct morphologies were selected, purified by streaking, and preserved for further study.

A preliminary screening for antagonistic activity was conducted using the dual culture method. A 100 μL suspension of *S. scabies* X-1 (1 × 108 cfu/mL, prepared from a 14-day culture on OMA) was evenly spread on Gause’s No. 1 agar plates. Strain XM18-5 was activated on LB agar at 30°C for 24 h. A 5-mm diameter agar plug containing the bacterial lawn was punched using a sterile cork borer and placed in the center of the pathogen-seeded plate. Plates were incubated at 30°C for 7 days, and the diameter of the inhibition zone was measured.

A secondary screening was performed using the Oxford cup method to evaluate the extracellular antimicrobial activity. Strains showing strong antagonism were activated and inoculated into 100 mL of LB broth (initial inoculum: 1% v/v of 1 × 108 cfu/mL suspension). The culture was incubated at 30°C with shaking at 180 rpm. To determine the optimal sampling time, a growth curve was constructed by measuring optical density (OD_600_) and viable count (cfu/mL) every 4 h. Based on the growth curve, fermentation broth was collected at 48 h. The culture was centrifuged at 12,000 rpm for 35 min, and the supernatant was filtered through a 0.22 μm sterile membrane to ensure it was cell-free. Sterile Oxford cups were placed on Gause’s No. 1 agar plates previously seeded with pathogen X-1. Each cup was filled with 200 μL of the cell-free supernatant. Sterile LB broth served as a negative control. The plates were incubated at 30°C for 7 days, and the diameter of the inhibition zone was measured.

### 2.3 Identification of the antagonistic strain XM18-5

#### 2.3.1 Morphological and physiological-biochemical identification

Strain XM18-5 was streaked onto an LB agar plate and incubated at 30°C for 24 h. Colony characteristics, including morphology, color, size, margin, and opacity, were recorded. Gram staining was performed for microscopic observation. Preliminary identification was based on Bergey’s Manual of Determinative Bacteriology. Physiological and biochemical tests, such as catalase activity, Voges-Proskauer test, starch hydrolysis, gelatin liquefaction, and nitrate reduction, were conducted using the HBI Bacillus identification kit (HBIG14).

#### 2.3.2 Molecular identification

Total genomic DNA of strain XM18-5 was extracted using the Solarbio bacterial genomic DNA kit. The 16S rDNA was amplified using universal primers 27F/1492R, respectively (Shrestha et al., 2015). The PCR products were sequenced by Sangon Biotech (Shanghai) Co., Ltd. The resulting sequences were subjected to a BLAST search against the NCBI GenBank database. Phylogenetic trees were constructed using the Neighbor-Joining method in MEGA 11.0 software with closely related sequences. 16S sequence data are deposited in the NCBI GenBank database under the accession number OK560566.

### 2.4 Whole-genome sequencing and bioinformatic analysis of XM18-5

Under sterile conditions, a single colony of XM18-5 was inoculated into a liquid medium and incubated at 30°C with shaking for 36 h. Bacterial cells were collected by centrifugation at 8,000 rpm for 5 min and washed three times with sterile water. Genomic DNA was extracted using the Solarbio Bacterial Genomic DNA Kit according to the manufacturer’s instructions. DNA quality and concentration were assessed using 1% agarose gel electrophoresis and a NanoDrop 2000 spectrophotometer (Thermo Scientific, United States). Whole genome sequencing was performed using the Illumina NovaSeq platform and PacBioRSII technology. Data analysis was conducted on the Majorbio Cloud Platform. Genome assembly was performed using SOAPdenovo2 (version 2.04) and sequence quality was verified using FastQC (version 0.11.9). Gene prediction was conducted using Glimmer (version 3.02). Functional annotation was performed by blasting genes against databases including NR, Swiss-prot, Pfam, COG, GO, and KEGG. Additionally, DNA sequence data are deposited in the NCBI GenBank database under the accession number CP199399.

Comparative genomic analysis of XM18-5, *B. velezensis* Ba-0321, DSM7, K-9, and SQR9 was performed using MUMmer (version 3.0) (Kurtz et al., 2004) and Mauve (version 2.3.1) (Darling et al., 2010) to analyze synteny. Pan-genome analysis and Venn diagram construction were performed using the EDGAR software framework (Blom et al., 2009).

### 2.5 Metabolomic analysis

#### 2.5.1 Sample preparation

Based on the growth curve analysis, samples were collected during the stationary phase to maximize secondary metabolite accumulation. Single colonies were inoculated into LB liquid medium and incubated at 28°C with 180 rpm shaking for 3 days (72 h). Bacterial cells were collected by centrifugation (5,000 rpm, 5 min, 4°C), washed 2–3 times with pre-cooled PBS, and the supernatant was completely discarded. The cell pellets were collected in 2 mL cryovials, snap-frozen in liquid nitrogen for 15 min, and stored at −80°C until analysis. Following this, the samples were dispatched to Biomarker Technologies for untargeted metabolomics analysis.

#### 2.5.2 LC-MS/MS analysis

The LC/MS system for metabolomics analysis is composed of Waters Acquity I-Class PLUS ultra-high performance liquid tandem Waters Xevo G2-XS QTof high resolution mass spectrometer. The column used is purchased from Waters Acquity UPLC HSS T3 column (1.8 μm 2.1*100 mm). Positive ion mode: mobile phase A: 0.1% formic acid aqueous solution; mobile phase B: 0.1% formic acid acetonitrile. Negative ion mode: mobile phase A: 0.1% formic acid aqueous solution; mobile phase B: 0.1% formic acid acetonitrile. Injection volume 2 μL.

#### 2.5.3 Data processing and analysis

The raw data collected using MassLynx V4.2 is processed by Progenesis QI software for peak extraction, peak alignment and other data processing operations, based on the Progenesis QI software online METLIN database and Biomark’s self-built library for identification. After normalizing the original peak area information with the total peak area, the follow-up analysis was performed. Principal component analysis and Spearman correlation analysis were used to judge the repeatability of the samples within group and the quality control samples. The identified compounds are searched for classification and pathway information in KEGG, HMDB, and lipidmaps databases. According to the grouping information, calculate and compare the difference multiples, T test was used to calculate the difference significance *p*-value of each compound. The R language package ropls was used to perform OPLS-DA modeling, and 200 times permutation tests was performed to verify the reliability of the model. The VIP value of the model was calculated using multiple cross-validation. The method of combining the difference multiple, the P value and the VIP value of the OPLS-DA model was adopted to screen the differential metabolites. The screening criteria are FC > 1, *P* < 0.05 and VIP > 1. The difference metabolites of KEGG pathway enrichment significance were calculated using hypergeometric distribution test.

### 2.6 Microscopic observation of antagonistic interaction

The effect of strain XM18-5 on the hyphal morphology of *S. scabies* X-1 was observed using Scanning Electron Microscopy (SEM). Samples were collected from the interaction zone of a dual culture plate. The samples were fixed overnight in 2.5% glutaraldehyde at 4°C, followed by dehydration through a graded ethanol series, critical-point drying, and gold sputtering. The specimens were then observed and photographed with an SEM.

For the observation of *S. scabies* X-1 morphology (interaction zone), samples were taken from the junction of the inhibition zone (approx. 5 mm from the XM18-5 colony) using the method described above. For the observation of pure *B. velezensis* XM18-5 morphology (Figure 3C), bacterial cells were cultured on sterile glass slides inserted into LB agar, incubated at 30°C for 12 h, fixed with 2.5% glutaraldehyde, and observed.

### 2.7 Antifungal spectrum assay

The broad-spectrum antifungal activity of strain XM18-5 was tested against ten common plant pathogenic fungi (see Table 1) using the dual culture method (Cui et al., 2022). A 5-mm mycelial plug of the pathogen was placed at the center of a PDA plate. A sterile filter paper disc (5 mm) was placed 2.5 cm away from the plug, and 3 μL of XM18-5 culture was applied to the disc. The plates were incubated in the dark at 30°C for 5–7 days. The inhibition rate was calculated using the formula: Inhibition (%) = [(Diameter of control colony - Diameter of treated colony)/Diameter of control colony] × 100.

**TABLE 1:** Pathogenic fungi for antifungal activity testing.

No.	Crop disease	Pathogen (scientific name)
1	Potato Early Blight	*Alternaria solani*
2	Potato Fusarium Wilt	*Fusarium oxysporum*
3	Potato Dry Rot	*Fusarium sambucinum*
4	Potato Anthracnose	*Colletotrichum coccodes*
5	Potato Gray Mold	*Botrytis cinerea*
6	Goji Berry Root Rot	*Fusarium oxysporum*
7	Maize Ear Rot	*Fusarium graminearum*
8	Grape Gray Mold	*Botrytis cinerea*
9	Maize Stalk Rot	*Fusarium verticillioides*
10	Watermelon Fusarium Wilt	*Fusarium oxysporum* f. sp. *niveum*

All strains used for the antifungal spectrum assay are maintained in the culture collection of the Ningxia Key Laboratory of Plant Disease and Pest Control.

### 2.8 Safety assay on potato tubers

Healthy, unblemished potato tubers were surface-sterilized with 75% ethanol, rinsed with sterile water, and sliced into 0.5-cm thick discs. The discs were immersed in a culture suspension of strain XM18-5 (1 × 108 cfu/mL) for 1 min. The treated discs were then placed in Petri dishes containing sterile moist filter paper. Discs treated with sterile water and LB broth served as controls. The plates were incubated at 30°C for 3–5 days to observe any signs of rot.

### 2.9 Biocontrol efficacy in pot experiment

Potato seed tubers were cut into 20–25 g blocks and planted in 25-cm diameter pots (three blocks per pot). When the plants reached the four-leaf stage, treatments were applied. Three groups were established: (1) Control: irrigated with 200 mL of water; (2) Pathogen: irrigated with 100 mL of *S. scabies* X-1 suspension (1 × 10^9^ cfu/mL) followed by 100 mL of water; (3) Biocontrol: irrigated with 100 mL of *S. scabies* X-1 suspension (1 × 10^9^ cfu/mL) followed by 100 mL of XM18-5 culture (1 × 10^6^ cfu/mL). Each treatment was replicated three times. At harvest, the disease incidence and disease index were recorded, and the control efficacy was calculated (Dees and Wanner, 2012).

### 2.10 Statistical analysis

All experimental data were analyzed using DPS 17.0 or SPSS 22.0 software. One-way analysis of variance (ANOVA) followed by Duncan’s multiple range test was used to compare means. A *P* < 0.05 was considered statistically significant.

## 3 Results

### 3.1 Isolation and screening of antagonistic strains

**FIGURE 1:**
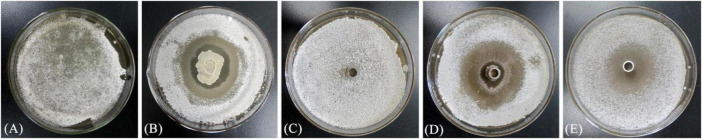
Bacteriostatic effect of strain XM18-5. **(A)** Primary screening control. **(B)** Primary screening effect. **(C)** Re-screening control. **(D)** Re-screening effect. **(E)** Inhibitory effect of the culture supernatant.

From 53 rhizosphere soil samples collected in Guyuan City, Ningxia Province, a total of 174 bacterial strains were isolated using the soil dilution plate method. Initial screening by the plate confrontation method identified 34 strains with significant antagonistic activity against *Streptomyces scabies* X-1. Subsequent rescreening using the Oxford cup method revealed that strain XM18-5, isolated from soil in Malian Township, Xiji County, exhibited strong inhibitory activity against X-1, with an inhibition zone diameter of 45.30 mm and an inhibition rate of 60.72%. Furthermore, the culture supernatant of XM18-5 displayed an inhibition zone diameter of 34.30 mm, corresponding to an inhibition rate of 45.98% (Figure 1). This indicates that XM18-5 is a strain with strong antagonistic potential.

**FIGURE 2:**
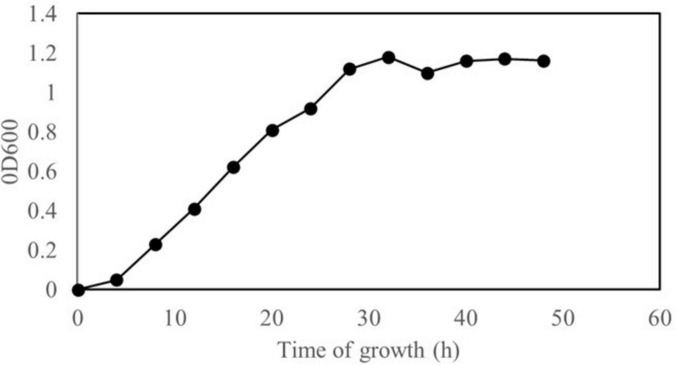
Growth curve of *B. velezensis* XM18-5.

The growth curve analysis indicated that strain XM18-5 entered the logarithmic growth phase at 4 h and reached the stationary phase plateau at approximately 36 h at 30°C (Figure 2). Therefore, supernatants collected at 36 h were used for antimicrobial activity assays.

### 3.2 Identification of strain XM18-5

#### 3.2.1 Morphological identification

**FIGURE 3:**
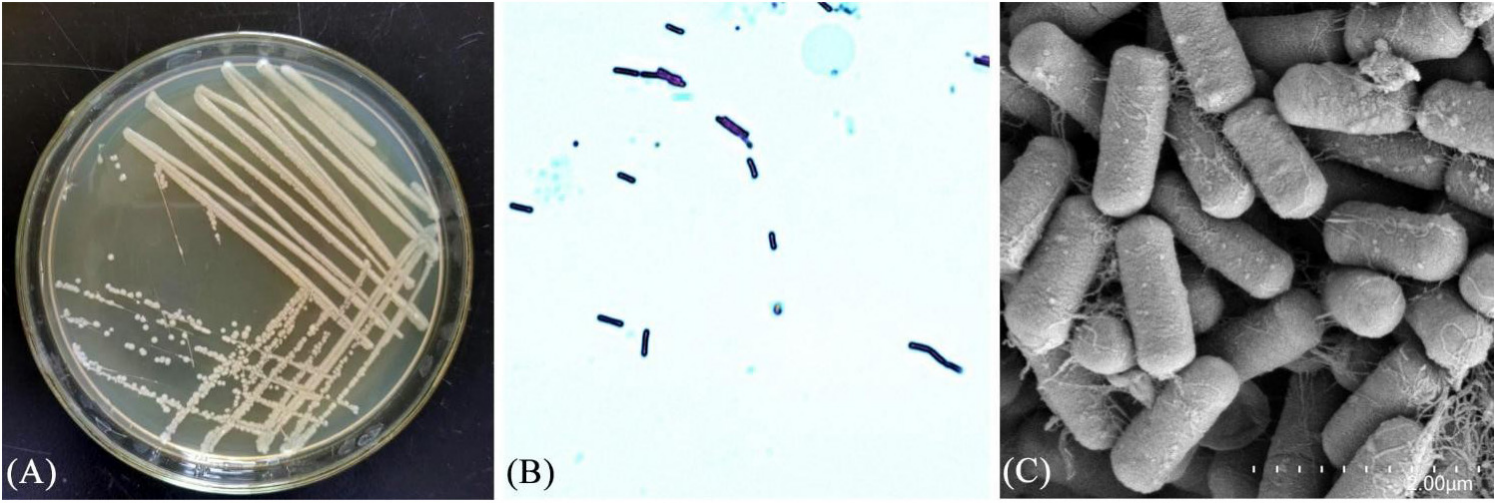
Morphological observation of strain XM18-5. **(A)** Colony morphology. **(B)** Microscopic results of Gram stain. **(C)** Morphology of electron microscopy scans.

Strain XM18-5 grew well on LB solid medium, forming milky white, opaque, slightly convex colonies with a slightly wrinkled surface and irregular edges, with no pigment precipitation around the colonies (Figure 3A). Microscopic examination showed Gram-positive staining (Figure 3B), and scanning electron microscopy revealed rod-shaped cells with a smooth surface and no shrinkage (Figure 3C). Based on these morphological characteristics, strain XM18-5 was preliminarily identified as belonging to the genus Bacillus.

#### 3.2.2 Physiological and biochemical characteristics identification

Physiological and biochemical tests showed that strain XM18-5 was positive for catalase and oxidase activity, capable of utilizing citrate, propionate, D-xylose, L-arabinose, glucose, and sucrose. It was positive for V-P and nitrate reduction reactions, grew normally at pH 5.7 and in 7% NaCl medium, hydrolyzed starch and gelatin, but could not utilize D-mannitol, maltose, or lactose, and produced urease but not esterase (Table 2). Combining these results with morphological characteristics and references such as Bergey’s Manual of Systematic Bacteriology, strain XM18-5 was preliminarily identified as belonging to the genus Bacillus. Further reference to Bacillus: volume 2, Bacillus Systematics, allowed its identification as *B. velezensis*.

**TABLE 2:** Physiological and biochemical characteristics of strain XM18-5.

Test index	Results
Catalase	+
Oxidase	+
V-P	+
Citrate	+
Propionate	+
D-Xylose	+
L-Arabinose	+
D-Mannitol	–
Gelatin liquefaction	+
7% NaCl	+
Growth at pH 5.7	+
Nitrate reduction	+
Starch hydrolysis	+
Maltose	–
Glucose	+
Sucrose	+
Lactose	–
Urease	+
Esterase	–

+: Positive; –: Negative.

#### 3.2.3 Molecular biology identification

**FIGURE 4:**
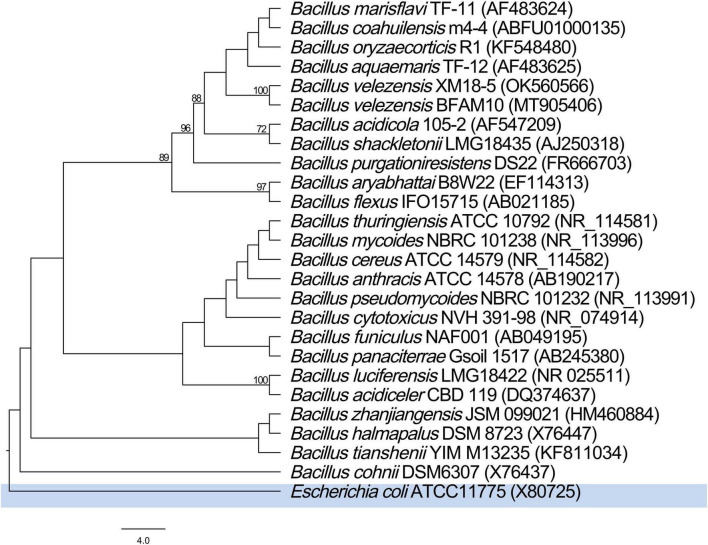
Phylogenetic tree based on 16S rDNA gene sequences, showing the evolutionary position of Bacillus velezensis XM18-5. Bootstrap values, based on 1,000 replicates, range from 71 (lowest) to 100 (highest). *Escherichia coli* ATCC11775 was used as the outgroup to root the tree.

Based on 16S rDNA gene sequencing, a 1,446 bp sequence was obtained (GenBank accession number: OK560566). BLAST comparison showed similarity greater than 98% with strains in the genus Bacillus. Phylogenetic tree analysis indicated that XM18-5 had 100% similarity with *B. velezensis* BCRC 17467 and clustered in the same branch (Figure 4).

### 3.3 Whole-genome sequencing and bioinformatics analysis of strain XM18-5

#### 3.3.1 Genome composition

**FIGURE 5:**
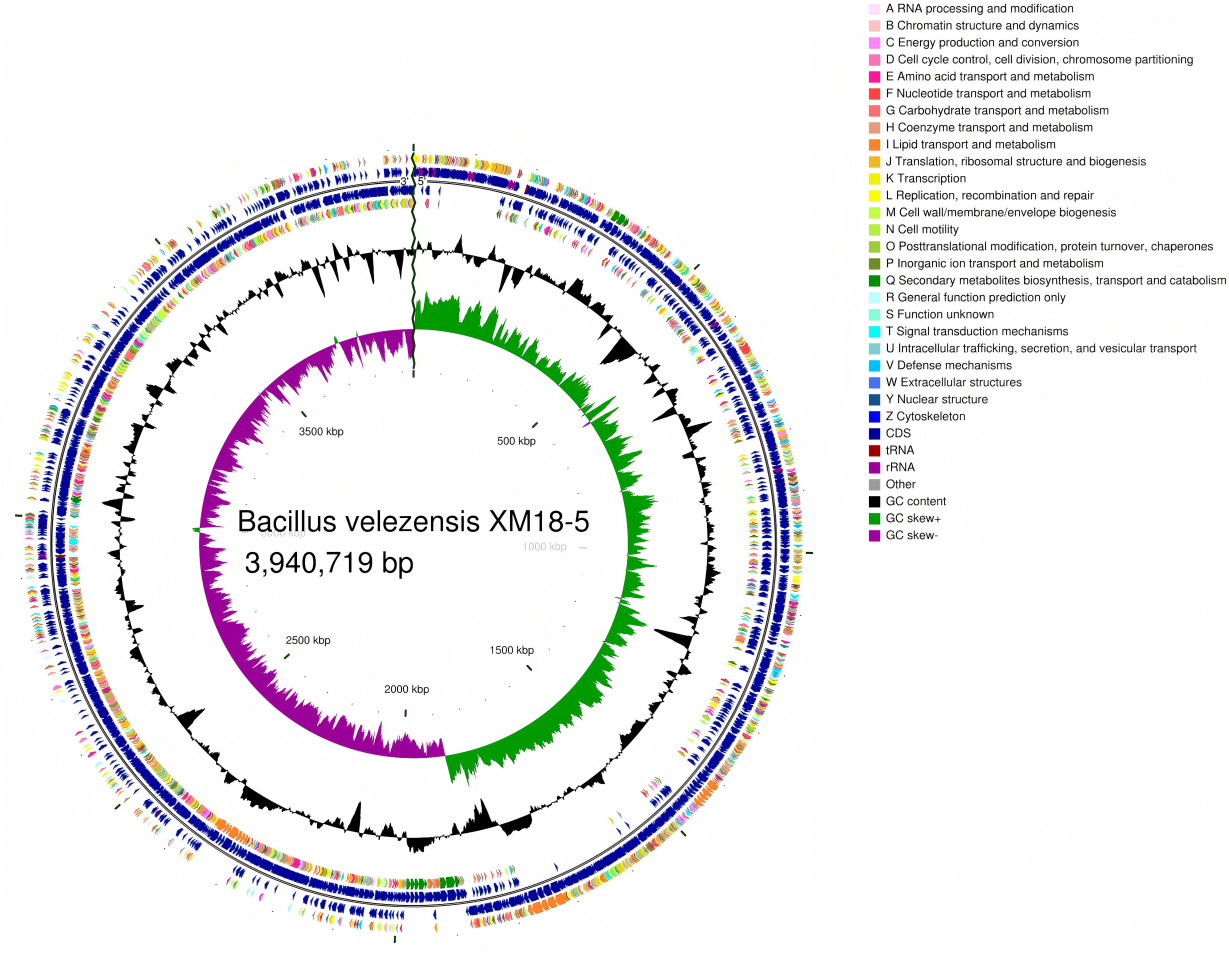
Genome circle map of XM18-5. From outer to inner circles: Rings 1 and 4: CDS on the positive and negative strands, respectively (colored by COG functional categories); Rings 2 and 3: CDS, tRNA, and rRNA on the positive and negative strands; Ring 5: GC content (outward peaks indicate higher than average GC, inward peaks indicate lower); Ring 6: GC skew (G-C/G+C, calculated to identify leading/lagging strands); Center: Genome size scale.

The complete genome of *Bacillus velezensis* XM18-5 was 3,940,719 bp in size with an average GC content of 46.49%. The genome contained 3,953 genes, including 3,759 protein-coding sequences (CDSs) with an average length of 926.49 bp. A total of 86 tRNA genes and 27 rRNA genes were identified, comprising nine copies each of 23S rRNA, 16S rRNA, and 5S rRNA genes. Additionally, 81 small RNA (sRNA) genes were predicted. Genomic analysis revealed three genomic islands, one prophage region, and 71 repeat sequences with a coverage of 0.43%. The genome sequencing data of strain XM18-5 were submitted to GenBank under accession number CP199399. The circular genome map is shown in Figure 5.

#### 3.3.2 Genome functional annotation

Functional annotation of the XM18-5 genome was performed using Diamond software (*E* ≤ 1e-5) by comparison against multiple databases including NR, Swiss-Prot, Pfam, EggNOG, GO, KEGG, CAZy, antiSMASH, VFDB, CARD, PHI, and TCDB. The alignment results with the highest scores were selected as annotation results. A total of 3,756 genes were successfully annotated, and the summary data are presented in Table 3. The top five databases by number of annotated genes were NR (3,756 genes, 99.9%), Swiss-Prot (3,536 genes, 94.1%), Pfam (3,355 genes, 89.3%), COG (3,084 genes, 82.0%), and GO (2,381 genes, 63.3%). The CAZy and CARD databases showed the lowest number of annotated genes with 129 (3.4%) and 290 (7.7%) genes, respectively.

**TABLE 3:** Distribution of gene functional annotation databases for strain XM18-5.

Type	Gene number	Annotation ratio
Total gene number	3,759	100%
NR	3,756	99.9%
Swiss-Prot	3,536	94.1%
Pfam	3,355	89.3%
COG	3,084	82.0%
GO	2,381	63.3%
KEGG	22,94	61.0%
PHI	814	21.7%
TCDB	768	20.4%
AntiSMASH	482	12.8%
VFDB	468	12.5%
CARD	290	7.7%
CAZy	129	3.4%

#### 3.3.3 GO functional annotation

**FIGURE 6:**
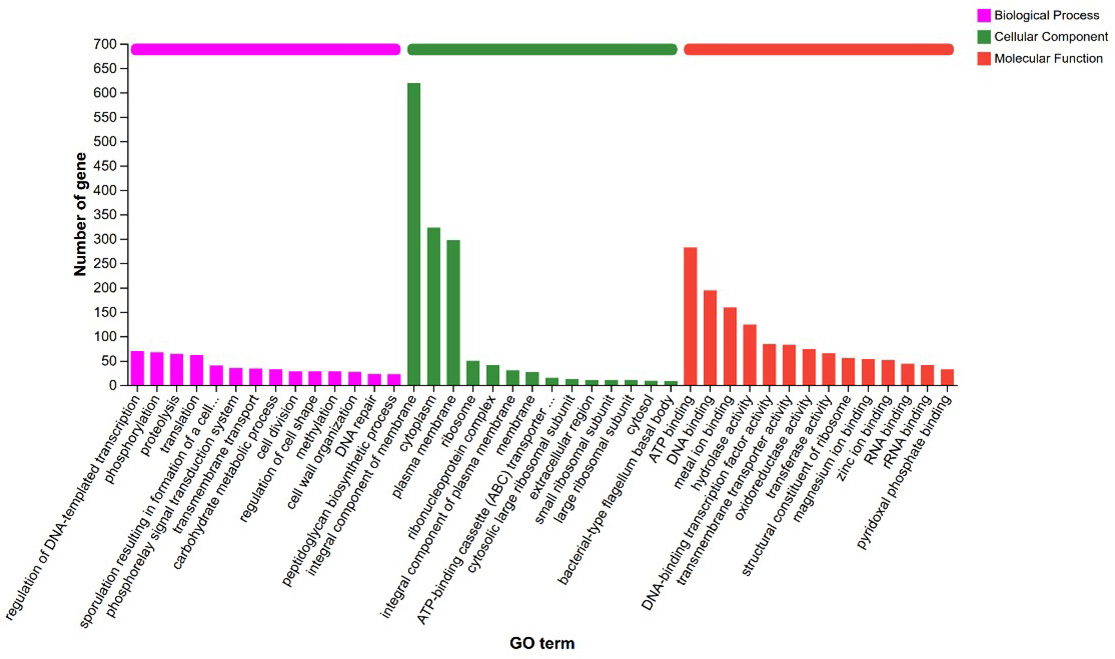
Gene ontology classification.

Comparison of the amino acid sequences of strain XM18-5 against the GO database resulted in the annotation of 2,294 protein-coding genes (58.03% of total genes), which were classified into three main categories: Biological Process (BP), Cellular Component (CC), and Molecular Function (MF) (Figure 6). In the BP category, three genes were annotated to the antibiotic biosynthetic process classification. In the MF category, 127 genes were annotated to hydrolase activity. Additionally, strain XM18-5 contained genes related to antagonistic activities, including one cellulase activity gene, two chitin binding protein genes, and genes associated with induced disease resistance, including one lipopolysaccharide biosynthetic gene, one acetolactate decarboxylase gene, and two acetolactate synthase genes. These results indicated that strain XM18-5 could function through multiple mechanisms including antibiotic production, enzyme secretion, and induction of plant disease resistance.

#### 3.3.4 COG functional annotation

**FIGURE 7:**
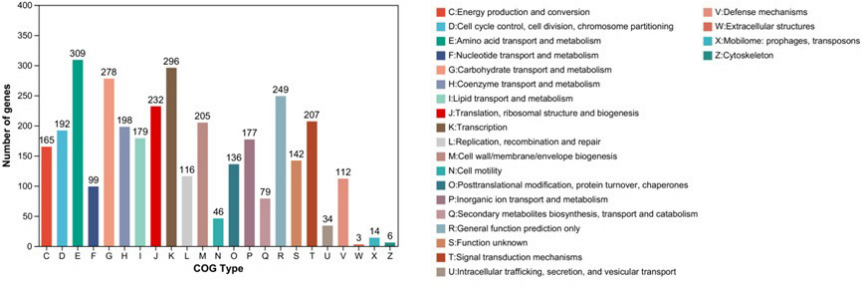
Classification diagram of COG results.

COG annotation of the biologically functional protein-coding genes in the XM18-5 genome revealed that 3,084 genes (78.02% of total genes) were annotated and classified into 23 categories (C to X and Z) (Figure 7). The largest category was amino acid transport and metabolism with 309 genes (10.02% of total annotated genes), followed by transcription (296 genes, 9.60%), carbohydrate transport and metabolism (278 genes, 9.01%), and general function prediction only (249 genes, 8.07%). Notably, 112 genes were annotated to defense mechanisms, with 32 genes encoding ABC-type multidrug transport systems being the most abundant. Additionally, seven genes each encoded ribonucleases and multidrug transporters, and two genes encoded non-specific conserved proteins involved in tellurite resistance. These genes were speculated to participate in the antimicrobial functions of strain XM18-5.

#### 3.3.5 KEGG functional annotation

**FIGURE 8:**
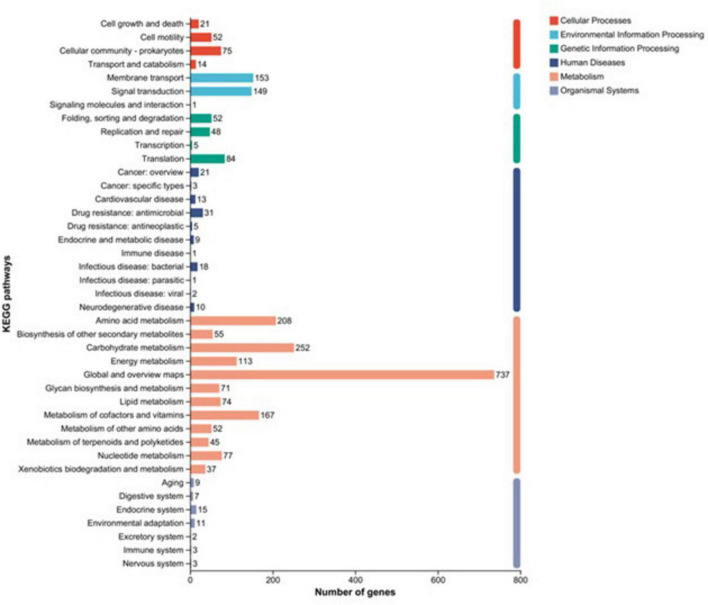
Classification diagram of KEGG pathway results.

Comparative analysis of strain XM18-5 against the KEGG database showed that 2,381 genes (60.23% of total genes) were mapped to KEGG pathways, participating in 41 metabolic pathways. Sixteen metabolic pathways contained more than 50 genes (Figure 8). KEGG enrichment analysis revealed that global and overview maps (737 genes), carbohydrate metabolism (252 genes), and amino acid metabolism (208 genes) were the three primary metabolic pathways. Furthermore, 55 genes were annotated to biosynthesis of other secondary metabolites, 56 genes to glycan biosynthesis and metabolism, 45 genes to metabolism of terpenoids and polyketides, and 37 genes to xenobiotics biodegradation and metabolism. These genes were speculated to be closely related to the production of antimicrobial substances by XM18-5.

#### 3.3.6 CAZy functional analysis

**FIGURE 9:**
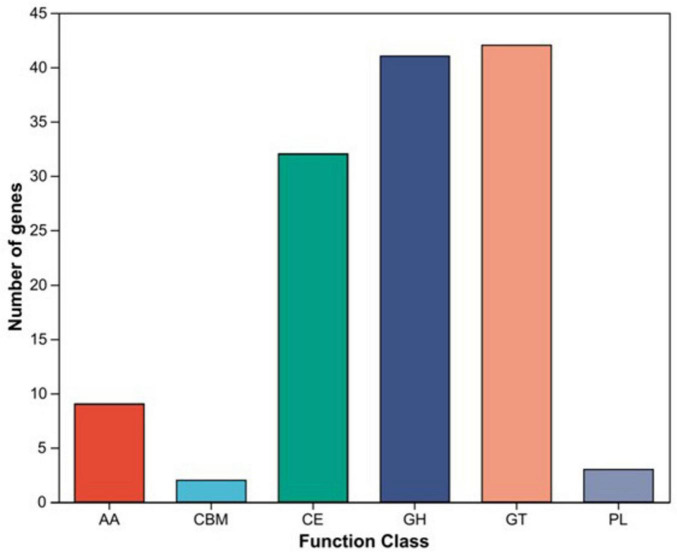
Statistical bar chart of CAZy database functional annotation for XM18-5 genome. AA, Auxiliary Activities; CBM, Carbohydrate-Binding Modules; CE, Carbohydrate Esterases; GH, Glycoside Hydrolases; GT, Glycosyl Transferases; PL, Polysaccharide Lyases.

Comparison of the genome sequence against the CAZy database revealed that 129 genes in the XM18-5 genome encoded protein domains belonging to CAZy families (Figure 9). These included 42 genes from 14 glycosyltransferase (GT) families, 41 genes from 28 glycoside hydrolase (GH) families, 32 genes from 9 carbohydrate esterase (CE) families, 9 genes from 6 auxiliary activity (AA) families, 3 genes from 3 polysaccharide lyase (PL) families, and 2 genes from 2 carbohydrate-binding module (CBM) families. The genome contained genes encoding endo-1,4-β-D-glucanase (EC 3.2.1.4), β-glucosidase (EC 3.2.1.21), and α-amylase (*amyE*, EC 3.2.1.1), which provide genetic evidence for understanding the biocontrol mechanisms of strain XM18-5.

#### 3.3.7 NR and Swiss-Prot database annotation

Translation of the gene sequences of strain XM18-5 into corresponding amino acid sequences and comparison against the NR database resulted in the annotation of 3,756 genes. The top 20 functions with clear annotation information are shown in Table 4. The Swiss-Prot database, a curated protein sequence database containing descriptions of protein function, structure, post-translational modifications, and variations, provided meaningful annotations for 3,536 genes in strain XM18-5.

**TABLE 4:** Top 20 distribution of NR database functional annotations for strain XM18-5 genome.

Type	Gene number	Annotation ratio
MULTISPECIES: MFS transporter	110	2.9%
MULTISPECIES: ABC transporter permease	105	2.8%
MULTISPECIES: ABC transporter ATP-binding protein	96	2.6%
MFS transporter	88	2.3%
MULTISPECIES: GNAT family N-acetyltransferase	85	2.3%
MULTISPECIES: response regulator transcription factor	70	1.9%
ABC transporter permease	70	1.9%
GNAT family N-acetyltransferase	66	1.8%
conserved hypothetical protein	60	1.6%
MULTISPECIES: MarR family transcriptional regulator	58	1.5%
ABC transporter ATP-binding protein	58	1.5%
MULTISPECIES: spore germination protein	43	1.1%
MULTISPECIES: LysR family transcriptional regulator	42	1.1%
MULTISPECIES: SDR family oxidoreductase	41	1.1%
MULTISPECIES: amino acid permease	37	1.0%
MULTISPECIES: TetR/AcrR family transcriptional regulator	35	0.9%
MarR family transcriptional regulator	35	0.9%
MULTISPECIES: tetratricopeptide repeat protein	32	0.9%
non-ribosomal peptide synthetase	31	0.8%
MULTISPECIES: helix-turn-helix domain-containing protein	31	0.8%

#### 3.3.8 Virulence factor and resistance gene analysis

**FIGURE 10:**
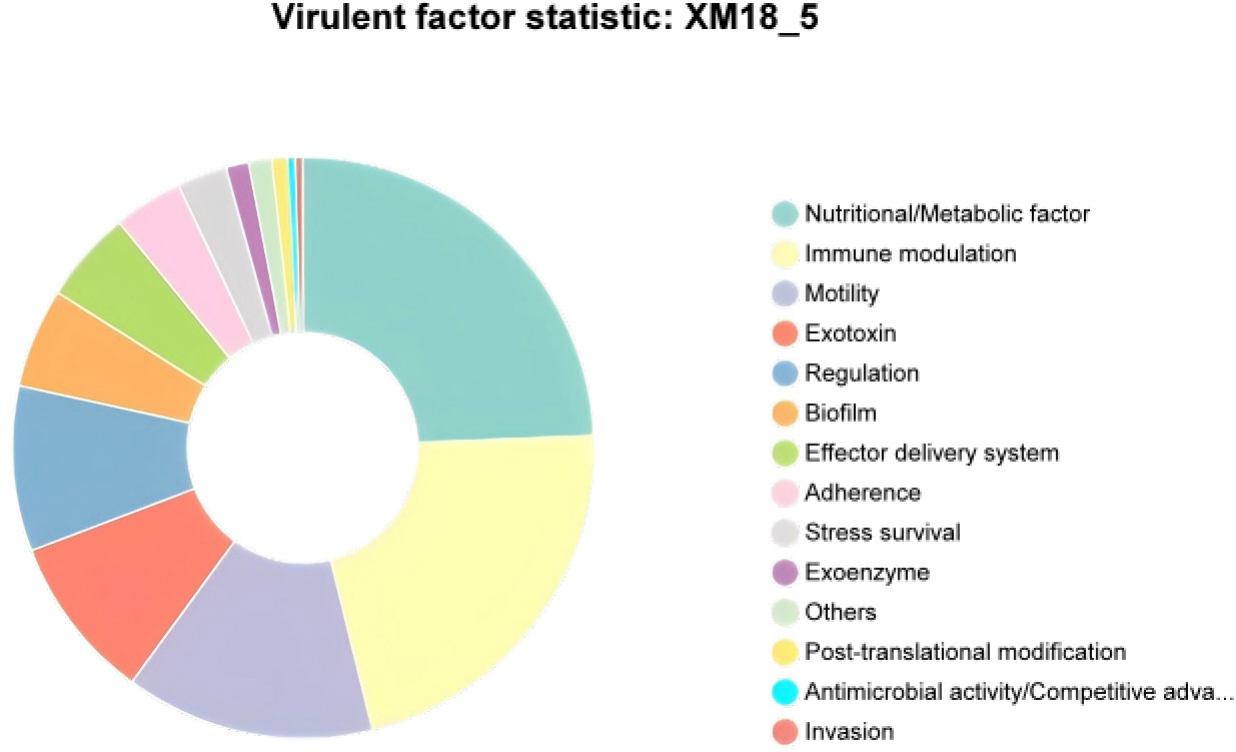
Statistical circular diagram of VFDB database functional annotation for XM18-5 genome.

Comparative analysis of the XM18-5 genome against the VFDB database identified 468 genes annotated to 14 major categories, 138 virulence factors, and 277 related functional genes (Figure 10 and Supplementary Table S5). This suggested that strain XM18-5 might release virulence factors to inhibit pathogen growth.

**FIGURE 11:**
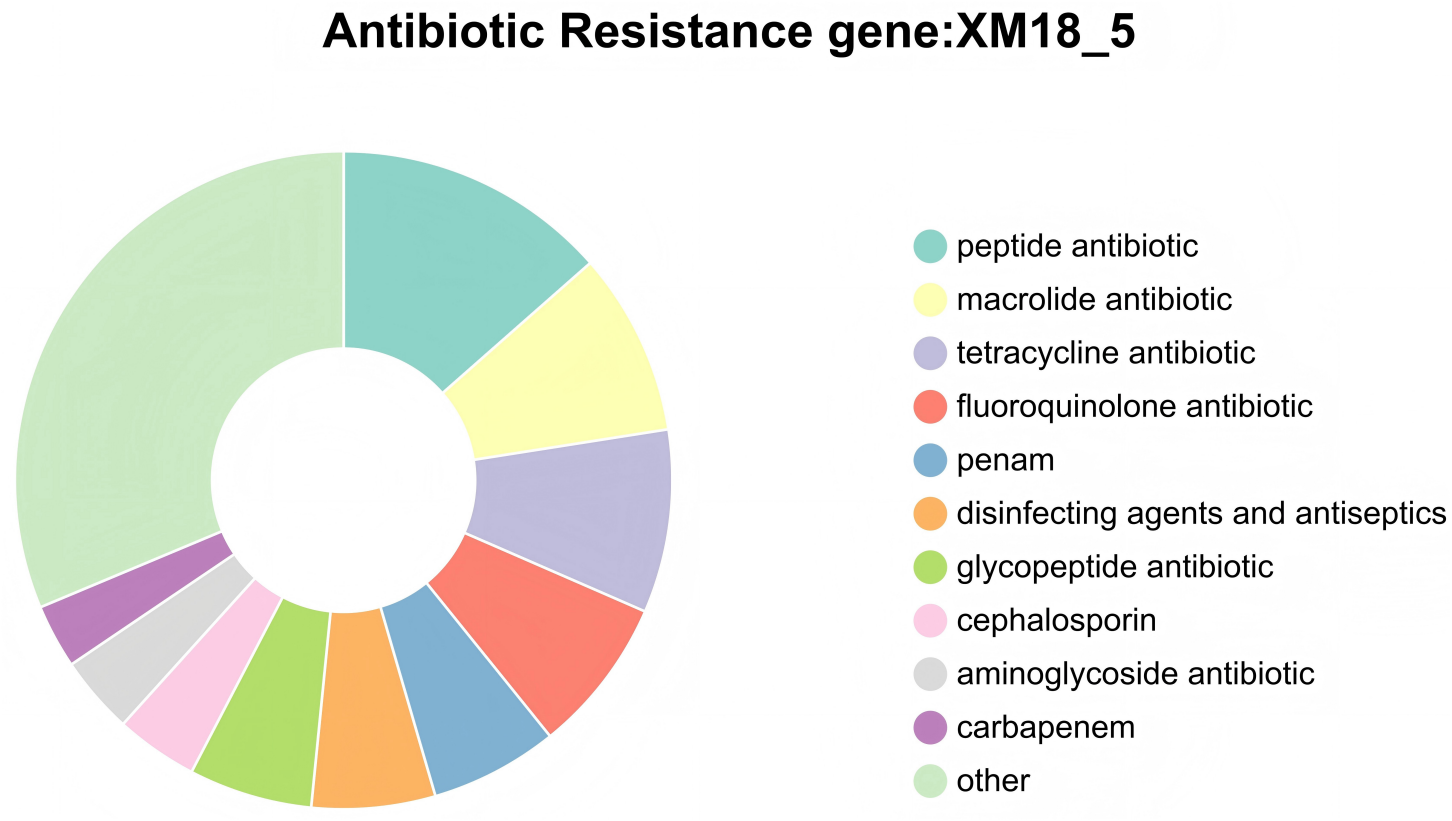
Statistical circular diagram of CARD database functional annotation for XM18-5 genome.

Annotation using the CARD database identified 290 genes in the XM18-5 genome, which were annotated to 38 drug classes comprising 159 Antibiotic Resistance Ontology (ARO) entries (Figure 11 and Supplementary Table S6). The top ten drug classes were peptide antibiotics, macrolide antibiotics, tetracycline antibiotics, fluoroquinolone antibiotics, penams, disinfecting agents and antiseptics, glycopeptide antibiotics, cephalosporins, aminoglycoside antibiotics, and cephamycins.

#### 3.3.9 Secondary metabolite biosynthesis prediction analysis

Analysis of the XM18-5 genome using antiSMASH revealed 12 secondary metabolite biosynthetic gene clusters. Six gene clusters showed complete identity or similarity above 90% to known clusters, one showed similarity below 20%, and four gene clusters had no similar known clusters identified (Table 5). Eight antimicrobial substances were identified: surfactin, butirosin, macrolactin H, bacillaene, fengycin, difficidin, bacillibactin, and bacilysin. Except for surfactin (82% similarity to BGC0000433) and butirosin (7% similarity to BGC0000693), the other six antibiotic biosynthetic gene clusters showed 100% similarity to corresponding clusters from known strains. Additionally, four gene clusters with unknown functions (Cluster 2, 3, 8, and 9) were identified, including two terpene clusters, one Type III PKS cluster, and one lanthipeptide-class-II cluster. These results indicated that strain XM18-5 harbored multiple gene clusters for producing antimicrobial secondary metabolites and potentially novel antimicrobial compounds, demonstrating significant biocontrol application potential.

**TABLE 5:** Gene clusters of secondary metabolite of XM18-5.

Gene cluster ID	Type	From	To	Similarity strain	Similarity known cluster	Similarity (%)
Cluster 1	NRPS	323,509	387,487	BGC0000433	Surfactin	82
Cluster 2	PKS-like	924,156	965,401	BGC0000693	Butirosin A/butirosin B	7
Cluster 3	Terpene	1,050,279	1,067,688	–	–	–
Cluster 4	Lanthipeptide-class-ii	1,188,677	1,217,566	–	–	–
Cluster 5	TransAT-PKS	1,384,185	1,472,021	BGC0000181	Macrolactin H	100
Cluster 6	TransAT-PKS	1,691,549	1,792,115	BGC0001089	Bacillaene	100
Cluster 7	NRPS	1,865,856	2,000,167	BGC0001095	Fengycin	100
Cluster 8	Terpene	2,028,804	2,050,688	–	–	–
Cluster 9	T3PKS	2,114,005	2,155,106	–	–	–
Cluster 10	TransAT-PKS	2,282,481	2,376,274	BGC0000176	Difficidin	100
Cluster 11	NRPS	3,012,021	3,063,813	BGC0000309	Bacillibactin	100
Cluster 12	Other	3,600,005	3,641,424	BGC0001184	Bacilysin	100

#### 3.3.10 Comparative genomics analysis

**FIGURE 12:**
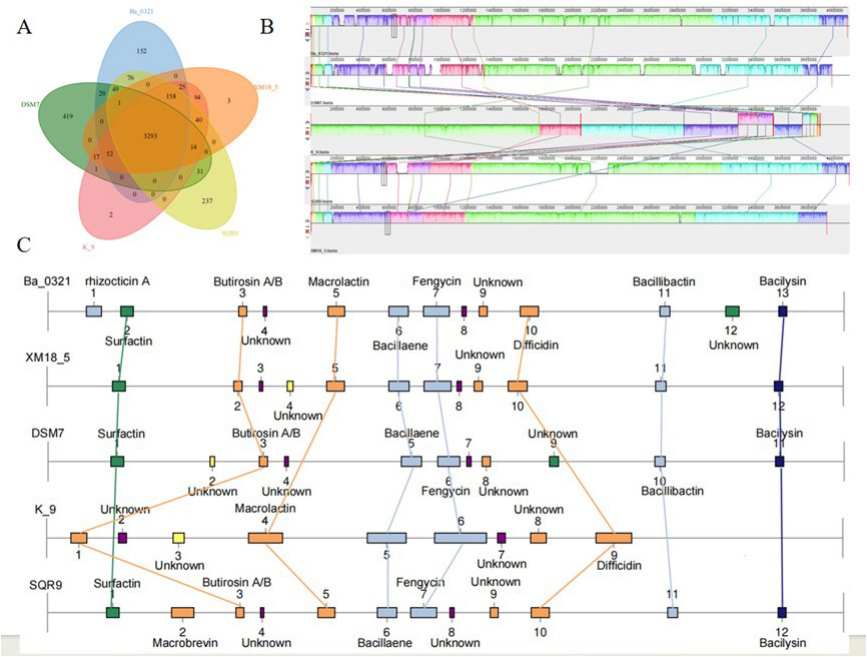
Comparative genomic analysis of strains XM18-5, Ba_0321, DSM7T, K-9 and SQR9. **(A)** Venn diagram of core, unique, and accessory genes among the strains, **(B)** Collinearity analysis with boxes of identical color indicating syntenic regions and rearrangements shown by colored lines, **(C)** Comparison of the locations and products of secondary metabolite gene clusters.

Comparison of genomic features between strain XM18-5 and five previously reported *B. velezensis* strains showed that the genome size, G+C content, and number of coding proteins of XM18-5 were similar to other *B. velezensis* genomes (Table 6). Homology analysis of core genes among XM18-5 and four other *B. velezensis* type strains (Ba_0321, DSM7T, K-9, and SQR9) revealed 3,293 shared core genes (Figure 12A), representing 88% of the total core genes in XM18-5 and primarily involved in basic life activities. Only three genes were unique to strain XM18-5. Furthermore, XM18-5 shared 3,693 core genes with K-9, suggesting functional similarity in certain core genes. Collinearity analysis of the five *B. velezensis* strains (Figure 12B) showed that most genes of XM18-5 exhibited direct linear correspondence with Ba_0321, DSM7T, K-9, and SQR9, although local inversions, translocations, and genome rearrangements were observed. Comparison of secondary metabolite biosynthetic gene cluster locations and products between XM18-5 and the other four *B. velezensis* strains revealed direct linear correspondence, consistent with the collinearity analysis results (Figure 12C).

**TABLE 6:** Comparison of genomic features of strain XM18-5 with other *B. velezensis* strains.

Strain	GenBank number	Genome size (Mb)	G+C content	Protein coding sequences	rRNA	tRNA	Isolation source	Reference
*B. velezensis* XM18-5	CP199399	3.94	46.5%	3759	27	86	Soil	This study
Ba-0321	CP101904.1	4.10	–	3839	27	86	Soil	(Li et al., 2023)
SQR9	CP006890.1	4.12	46.0%	3904	21	72	Cucumber rhizosphere	(Zhang et al., 2015)
DSM 7T	NC_014551.1	3.98	46.1%	3870	30	93	Laboratory	(Borriss et al., 2011)
K-9	JAKQYO000000000.1	3.89	46.5%	3680	–	–	Potato	(Shuang et al., 2022)

### 3.4 Metabolomic analysis

#### 3.4.1 Overall metabolic profile analysis of XM18-5 fermentation supernatant

**FIGURE 13:**
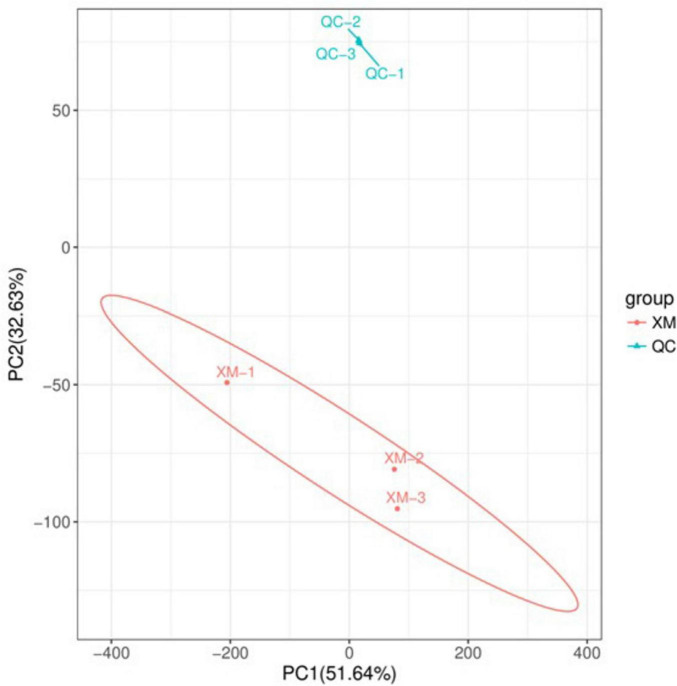
Principal Component Analysis (PCA) plot of metabolites from the *B. velezensis* XM18-5 fermentation supernatant.

Principal component analysis (PCA) of the untargeted metabolomics data showed good repeatability in the analysis, with clear sample grouping. Quality control samples clustered tightly, indicating high instrument and data stability. The three biological replicates of *B. velezensis* XM18-5 fermentation broth (XM-1, XM-2, XM-3) formed distinct clusters, primarily separated along PC1. PC1 and PC2 explained 51.64 and 32.63% of the total variance, respectively (cumulative 84.27%), indicating that the metabolic variation was mainly captured by the first two principal components, and the fermentation produced a consistent metabolic profile (Figure 13).

#### 3.4.2 Functional classification of differential metabolites

**FIGURE 14:**
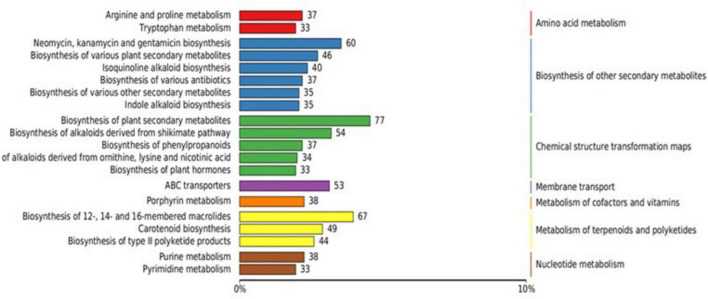
KEGG functional classification of the differential metabolites.

Upregulated differential metabolites were annotated and classified using the KEGG database, with most falling under the “Metabolism” superclass. Major subclasses included “Amino acid metabolism,” “Other amino acid metabolism,” “Carbohydrate metabolism,” and “Biosynthesis of other secondary metabolites.” This suggests that primary and secondary metabolic networks are highly active during *B. velezensis* XM18-5 fermentation, consistent with the characteristics of biocontrol Bacillus strains producing bioactive compounds (Figure 14).

#### 3.4.3 Metabolite annotation in biocontrol-related pathways

**FIGURE 15:**
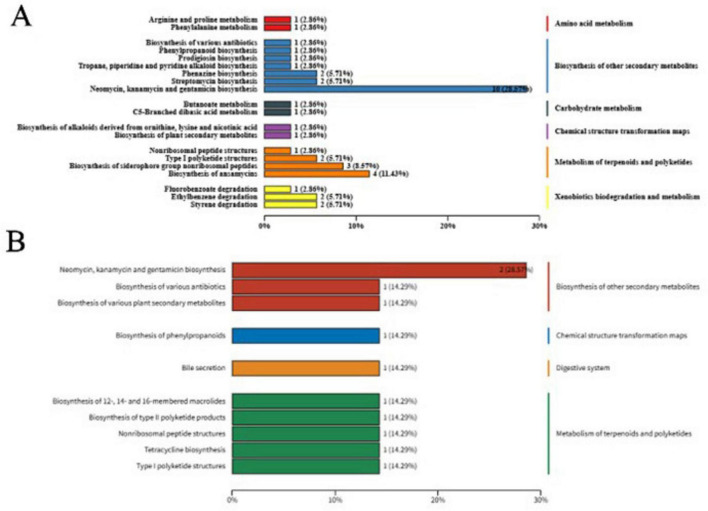
**(A)** Functional annotation of differential metabolites in biocontrol-related KEGG pathways. **(B)** Functional classification of identified antimicrobial metabolites in KEGG pathways.

Focusing on KEGG pathways related to biocontrol, differential metabolites were mainly annotated in “Biosynthesis of secondary metabolites,” “Biosynthesis of antibiotics,” and “Biosynthesis of amino acids.” Metabolites in amino acid biosynthesis pathways showed high abundance, serving as key precursors for antimicrobial lipopeptides such as fengycin and surfactin, supporting the metabolic basis of XM18-5’s antagonistic activity (Figure 15A).

To further pinpoint the chemical basis of the antimicrobial activity, we screened the metabolome for specific antimicrobial compounds. A total of 9 key antimicrobial metabolites were identified (Supplementary Table S1), including the lipopeptide Surfactin, the dipeptide antibiotic Bacilysin, and several broad-spectrum antibiotics such as Gentamicin X2, Tobramycin, and Erythromycin. The functional classification of these specific antimicrobial metabolites revealed that the majority were mapped to “Neomycin, kanamycin and gentamicin biosynthesis” (28.57%), followed by “Biosynthesis of various antibiotics,” “Biosynthesis of secondary metabolites,” and “Biosynthesis of phenylpropanoids” (each 14.29%). This specific annotation confirms that the secondary metabolic machinery of XM18-5 is actively directing resources toward diverse antibiotic synthesis pathways (Figure 15B).

#### 3.4.4 Enrichment analysis of key biocontrol-related metabolic pathways

**FIGURE 16:**
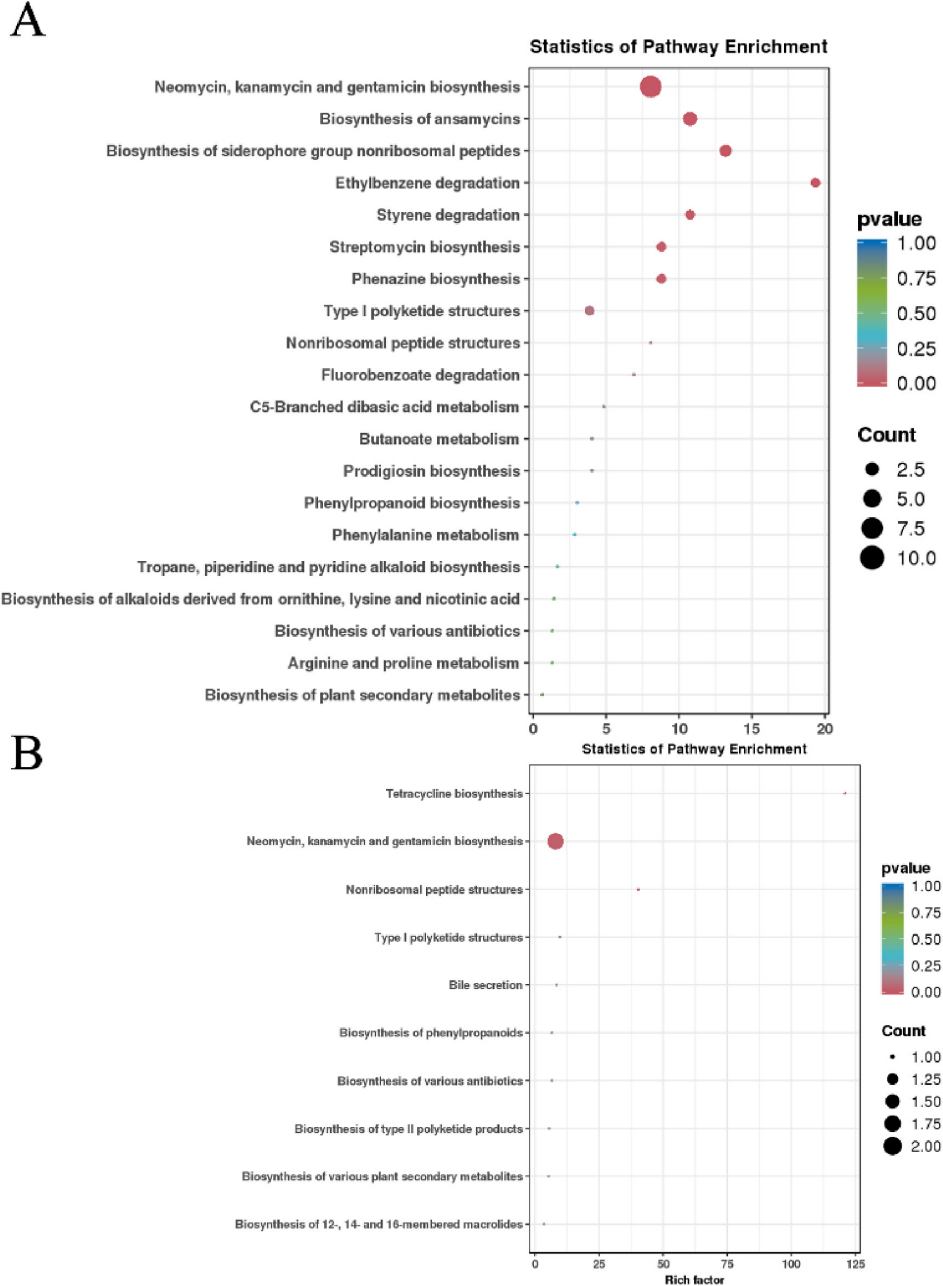
**(A)** KEGG pathway enrichment analysis of differential metabolites related to biocontrol. **(B)** KEGG pathway enrichment analysis of identified antimicrobial metabolites.

KEGG pathway enrichment analysis showed significant enrichment in pathways such as “Valine, leucine, and isoleucine biosynthesis” and “Phenylalanine, tyrosine, and tryptophan biosynthesis” (high Rich Factor and low q-value). Additionally, pathways like “Aminoacyl-tRNA biosynthesis” and “Amino acid biosynthesis” were highly enriched. Activation of these pathways indicates that the metabolic machinery of XM18-5 is strategically mobilized to supply precursors for the large-scale production of its antimicrobial compounds (Figure 16A).

Further enrichment analysis focused specifically on the identified antimicrobial metabolites revealed distinct pathway activities. The results indicated that “Biosynthesis of 12-, 14-, and 16-membered macrolides” and “Biosynthesis of various plant secondary metabolites” had the highest Rich Factors, suggesting a highly specialized synthesis of macrolide antibiotics (e.g., Erythromycin) and coumarins (e.g., Scopoletin). Furthermore, pathways such as “Biosynthesis of type II polyketide products” and “Nonribosomal peptide structures” were also enriched (Figure 16B). This targeted enrichment analysis corroborates the production of polyketides and non-ribosomal peptides (NRPs) like Surfactin, providing a clear metabolic signature for the strain’s potent antibacterial and antifungal efficacy.

### 3.5 Scanning electron microscopy observation of the effect of strain XM18-5 on the morphology of *Streptomyces scabies* X-1

**FIGURE 17:**
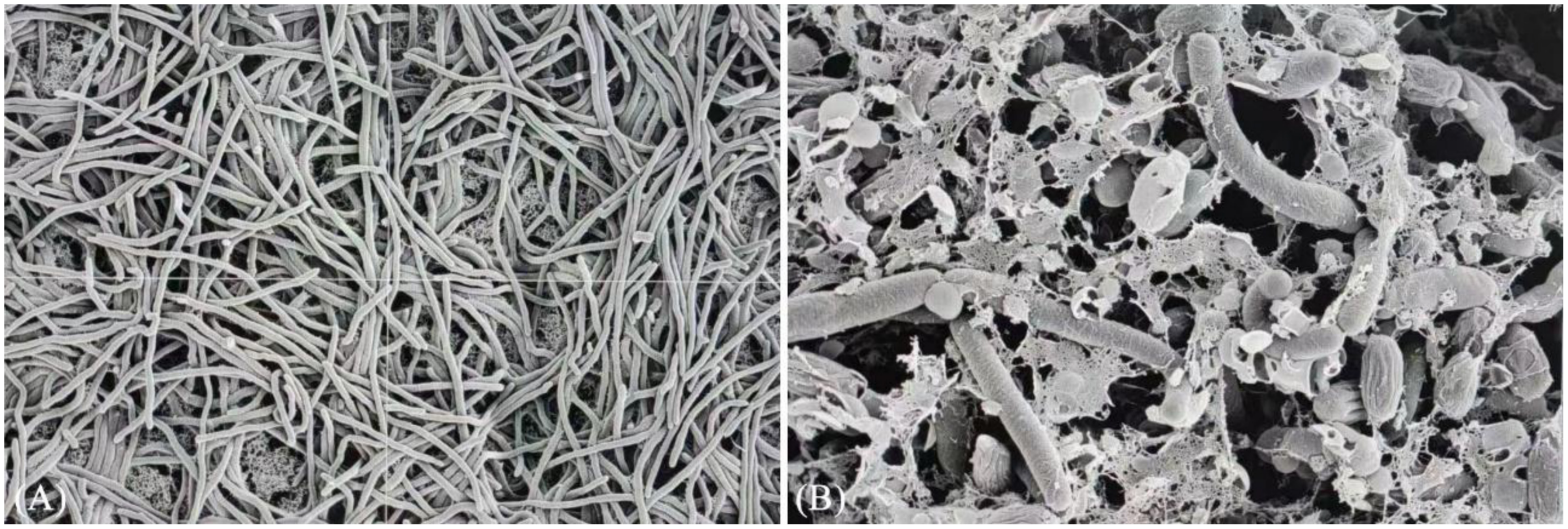
Effect of XM18-5 on the morphology of hyphae of *Streptomyces scabies* under scanning electron microscope. **(A)** *Streptomyces scabies* X-1. **(B)** Strain XM18-5 antagonizes *Streptomyces scabies*.

Results showed that XM18-5 inhibited the growth of X-1 hyphae. Normal X-1 hyphae had a smooth surface, while treated hyphae exhibited deformation, wrinkling, and cell wall lysis (Figure 17), indicating that XM18-5 has a direct destructive effect on the pathogen.

### 3.6 Antifungal spectrum assay of strain XM18-5

**FIGURE 18:**
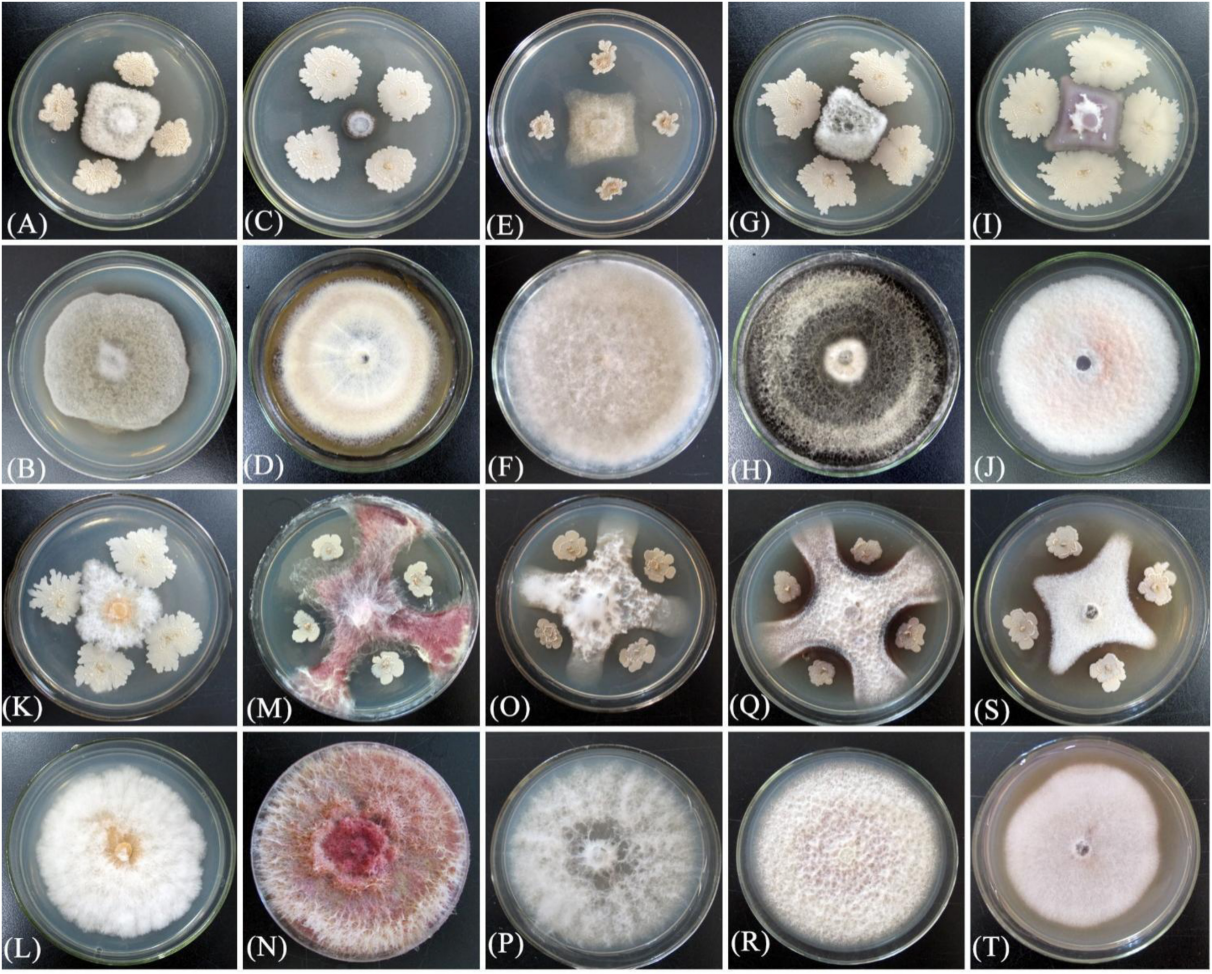
Antifungal activity of strain XM18-5 against ten plant pathogenic fungi. **(A)** Potato early blight fungus *Alternaria solani* experimental group. **(B)** Potato early blight fungus *Alternaria solani* control group. **(C)** Potato anthracnose fungus *Colletotrichum coccodes* experimental group. **(D)** Potato anthracnose fungus *Colletotrichum coccodes* control group. **(E)** Grape gray mold fungus *Botrytis cinerea* experimental group. **(F)** Grape gray mold fungus *Botrytis cinerea* control group. **(G)** Potato gray mold fungus *Botrytis cinerea* experimental group. **(H)** Potato gray mold fungus *Botrytis cinerea* control group. **(I)** Potato wilt fungus *Fusarium oxysporum* experimental group. **(J)** Potato wilt fungus *Fusarium oxysporum* control group. **(K)** Potato dry rot fungus *Fusarium sambucinum* experimental group. **(L)** Potato dry rot fungus *Fusarium sambucinum* control group. **(M)** Corn stalk rot fungus *Fusarium verticillioides* experimental group. **(N)** Corn stalk rot fungus *Fusarium verticillioides* control group. **(O)** Watermelon wilt fungus *Fusarium oxysporum f.* sp. niveum experimental group. **(P)** Watermelon wilt fungus *Fusarium oxysporum f.* sp. niveum control group. **(Q)** Corn ear rot fungus *Fusarium graminearum* experimental group. **(R)** Corn ear rot fungus *Fusarium graminearum* control group. **(S)** Wolfberry root rot fungus *Fusarium oxysporum* experimental group. **(T)** Wolfberry root rot fungus *Fusarium oxysporum* control group.

XM18-5 showed varying degrees of inhibition against 10 plant pathogens, with the highest inhibition rate against potato anthracnose fungus C. coccodes at 88.23%, significantly higher than others (P < 0.05). The average inhibition rates ranged from 54.25 to 88.23% (Table 7), demonstrating the broad-spectrum inhibitory potential of XM18-5 (Figure 18).

**TABLE 7:** Inhibition rate of strain XM18-5 against 10 pathogenic fungi.

Number	Plant pathogens	Average inhibition rate (%)
1	Potato early blight fungus *A. solani*	63.44 ± 1.00d
2	Potato anthracnose fungus *C. coccodes*	88.23 ± 0.88a
3	Grape gray mold fungus *B. cinerea*	71.67 ± 1.82b
4	Potato gray mold fungus *B. cinerea*	71.07 ± 0.55bc
5	Potato wilt fungus *F. oxysporum*	69.79 ± 0.57bc
6	Potato dry rot fungus *F. sambucinum*	67.27 ± 1.98cd
7	Corn stalk rot fungus *F. verticillioides*	65.42 ± 1.82d
8	Watermelon wilt fungus *F. oxysporum f.* sp. niveum	57.50 ± 0.72e
9	Corn ear rot fungus *F. graminearum*	56.25 ± 0.00e
10	Wolfberry root rot fungus *F. oxysporum*	54.25 ± 2.20e

Data are the average of three replicates, and the same lowercase letters indicate no significant difference at the 0.05 level.

### 3.7 Safety assay of strain XM18-5 on potato tubers

**FIGURE 19:**
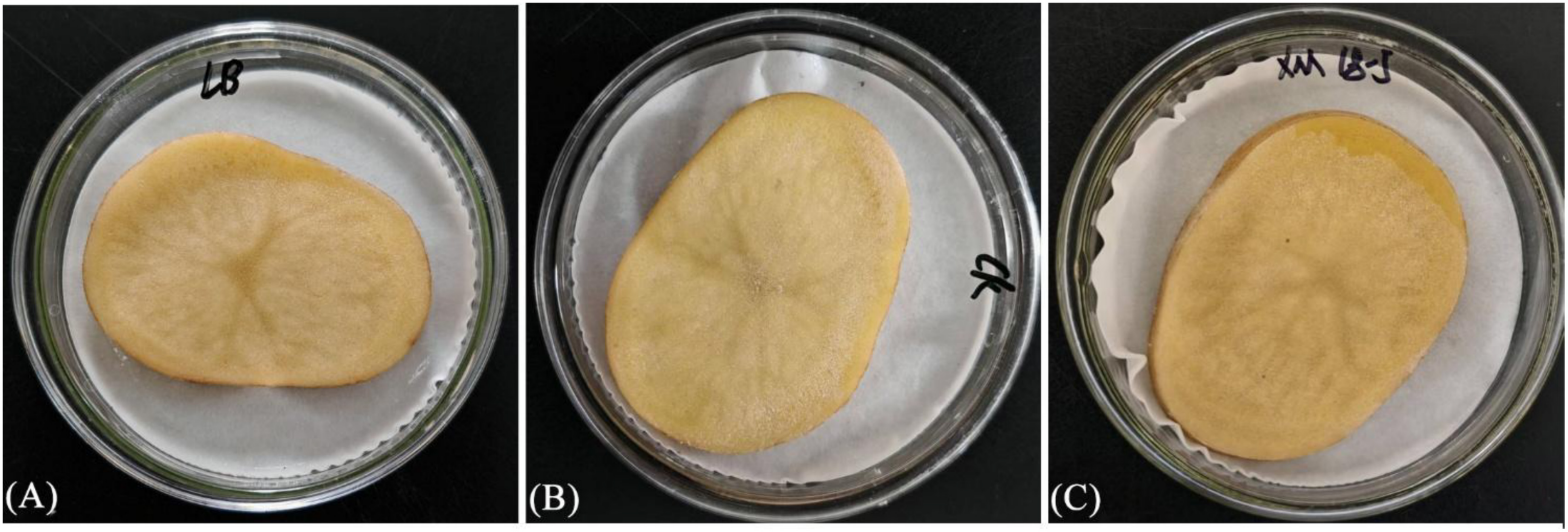
Safety testing of XM18-5 strain on potato chunks. **(A)** LB culture medium. **(B)** Sterile distilled water. **(C)** Strain XM18-5 culture medium.

Potato slices treated with XM18-5 culture at 1 × 10^8 cfu/mL showed no signs of rot after 5 days, with no significant difference from the control group, indicating that XM18-5 is safe for potatoes (Figure 19).

### 3.8 Pot experiment for biocontrol efficacy

**FIGURE 20:**
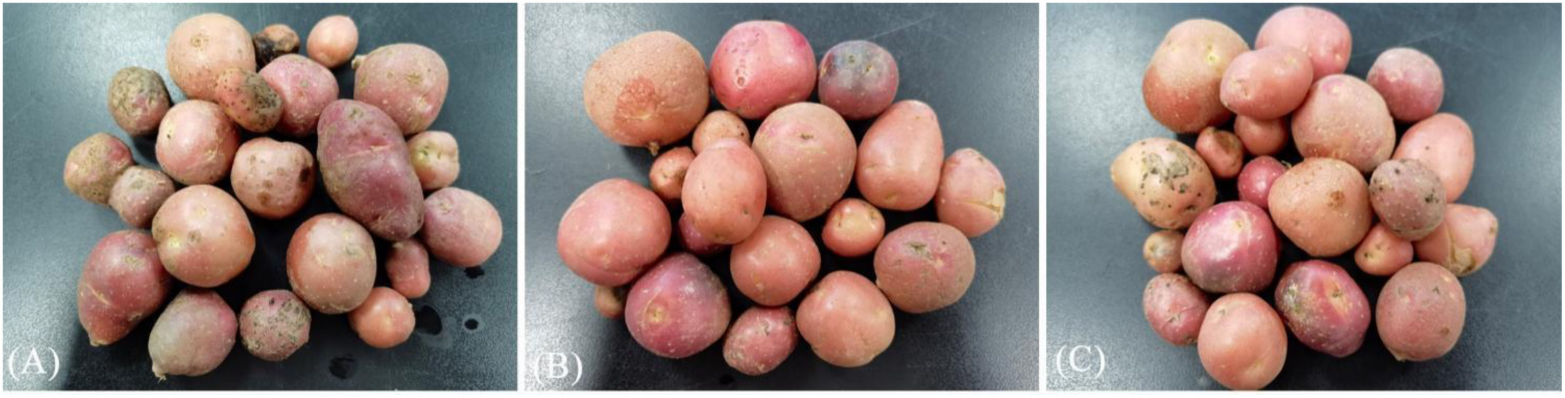
The preventive effect of strain XM18-5 on potato common scab. **(A)** Pathogen treatment group. **(B)** Water treatment group. **(C)** Biocontrol bacteria and pathogen treatment group.

Results showed that the pathogen-treated group had a potato incidence rate of 53.56% and a disease index of 49.50. The XM18-5-treated group had an incidence rate of 25.00% and a disease index of 14.40, with a preventive effect of 70.90% (Table 8). This demonstrates that XM18-5 can effectively control potato common scab (Figure 20).

**TABLE 8:** The biocontrol efficacy of strain XM18-5 on potato common scab.

Treatment	Occurrence rate (%)	Disease index (%)	Biocontrol efficacy (%)
1 (Control)	0	0	/
2 (Pathogen)	53.56 ± 1.20a	49.50 ± 2.30a	/
3 (XM18-5)	25.00 ± 1.40b	14.40 ± 1.45b	70.90 ± 0.40

The same lowercase letters indicate no significant difference at the 0.05 level.

## 4 Discussion

This study isolated and identified a potent biocontrol strain, *Bacillus velezensis* XM18-5, from soil infested with potato common scab. Phenotypic evaluations confirmed that the strain exhibits strong direct antagonism against *Streptomyces scabies*, achieving a control efficacy of up to 70.90% in pot experiments, and displays broad-spectrum inhibitory activity against various plant pathogens. To uncover the molecular mechanisms underlying its superior biocontrol effects, this research integrated genomics and metabolomics to elucidate the core mode of action by which XM18-5 inhibits *S. scabies*.

Genome mining revealed the formidable genetic potential of *B. velezensis* XM18-5 for biocontrol. Genomic analysis is a critical step in unveiling the mechanisms of action and assessing the application potential of biocontrol strains (Bertê et al., 2022; Lagzian et al., 2023). The genome size (3.94 Mb) and framework of strain XM18-5 are similar to those of previously reported high-efficacy biocontrol strains such as FZB42 and SQR9 (Chen et al., 2009; Zhang et al., 2015). Analysis with antiSMASH identified 12 secondary metabolite biosynthesis gene clusters (BGCs) within its genome, indicating a strong capacity for producing bioactive substances. These BGCs include those encoding non-ribosomal peptides (NRPs) like surfactin and fengycin, and polyketides (PKS) such as macrolactin H and difficidin. Notably, six of these BGCs share 100% similarity with known clusters, providing robust evidence for the genetic basis of XM18-5 to produce a diverse array of potent antimicrobial compounds (Wang et al., 2019; Sousa et al., 2025; Zhao et al., 2025).

Importantly, our metabolomic analysis successfully detected the presence of key antimicrobial compounds, validating the genomic predictions. Specifically, the lipopeptide Surfactin (m/z 1056.65) and the dipeptide Bacilysin (m/z 235.11) were identified in the fermentation broth. Crucially, the detection of Surfactin and Bacilysin provides a direct mechanistic explanation for the observed antagonism. Surfactin is well-known for its ability to disrupt pathogen cell membranes (Meena and Kanwar, 2015), which directly explains the severe deformation and rupture of *S. scabies* hyphae observed under scanning electron microscopy in this study. Furthermore, we detected a diverse range of antibiotics, including aminoglycosides (Gentamicin X2, Tobramycin), macrolides (Erythromycin), and tetracyclines (Tetracycline). The detection of Scopoletin, a coumarin derivative usually associated with plant defense, is also intriguing and suggests XM18-5 may synthesize or metabolize plant-associated compounds to enhance environmental fitness. Simultaneously, Bacilysin acts as a “Trojan horse” antibiotic; it is transported into pathogen cells and hydrolyzed to release anticapsin, which inhibits glucosamine-6-phosphate synthase, blocking cell wall synthesis (Wang et al., 2018). The dual attack on cell membranes (Surfactin) and cell walls (Bacilysin) constitutes a lethal synergistic strategy. Furthermore, the unexpected identification of broad-spectrum antibiotics, including macrolides (Erythromycin) and aminoglycosides, suggests that XM18-5 may have acquired or activated diverse pathway capabilities to maintain a competitive advantage in the complex soil microbiome.

Metabolomic analysis provided direct material evidence for the potential predicted by the genome. While the genome reveals the genetic potential of a strain, metabolomics elucidates its actual metabolic output and functional realization (Chen et al., 2025). The non-targeted metabolomic analysis in this study showed that “valine, leucine, and isoleucine biosynthesis” and “phenylalanine, tyrosine, and tryptophan biosynthesis” were the most significantly enriched metabolic pathways in the strain’s fermentation broth. These branched-chain and aromatic amino acids are essential precursors for the synthesis of antimicrobial lipopeptides like surfactin and fengycin (Wang et al., 2023). This is strongly supported by the specific detection of Surfactin in our samples, confirming that the upregulation of these amino acid pathways directly translates into the production of final bioactive lipopeptides. This finding indicates that the primary metabolic system of XM18-5 is preferentially mobilized to ensure an abundant supply for the downstream synthesis of antimicrobial lipopeptides. This forms a logical loop with the genomic functional annotations, where a large number of genes were enriched in “amino acid metabolism” and “biosynthesis of other secondary metabolites.” This complete chain of evidence, from genetic potential to metabolic readiness to functional performance, systematically clarifies the biocontrol mode of action of XM18-5.

In addition to direct antibiosis, XM18-5 possesses the genetic potential for other synergistic biocontrol mechanisms. The genome contains genes encoding 41 glycoside hydrolases (GHs) and chitin-binding proteins, which may act by degrading the chitin-rich cell wall of *Streptomyces* spp. (Wang et al., 2025). Furthermore, the identification of genes for acetolactate synthase and decarboxylase, key enzymes in the synthesis of volatile organic compounds (VOCs) like acetoin and 2,3-butanediol, suggests another mode of action. These VOCs are known to not only inhibit pathogens directly but also to induce systemic resistance (ISR) in the host plant (Ubhayasekera, 2011; Sivaramakrishnan et al., 2024). This suggests that XM18-5 may protect plants through multiple mechanisms, highlighting its potential as a multifunctional biocontrol agent.

In summary, *B. velezensis* XM18-5 is not merely a producer of a single antibiotic, but a sophisticated biological factory. Its high efficacy against potato common scab is driven by a “cocktail” mechanism: the genomic encoding and metabolic production of membrane-disrupting lipopeptides (Surfactin) and cell-wall-inhibiting antibiotics (Bacilysin), supported by a rewired primary metabolism that ensures efficient precursor supply. These findings provide a solid theoretical foundation for the formulation of XM18-5 as a novel, effective microbial fertilizer for sustainable potato production.

## Data Availability

16S sequence data are deposited in the NCBI GenBank database under the accession number OK560566. Additionally, DNA sequence data are deposited in the NCBI GenBank database under the accession number CP199399. The genome sequencing data of strain XM18-5 were submitted to GenBank under accession number CP199399.
